# Manganese-doping enhanced local heterogeneity and piezoelectric properties in potassium tantalate niobate single crystals

**DOI:** 10.1107/S2052252521000890

**Published:** 2021-02-24

**Authors:** Yu Wang, Peng Tan, Xiangda Meng, Zhongxiang Zhou, Xiaolin Huang, Chengpeng Hu, Fei Huang, Jing Wang, Hao Tian

**Affiliations:** a School of Physics, Harbin Institute of Technology, Harbin 150001, People’s Republic of China; b Key Laboratory of Micro-Nano Optoelectronic Information System, Ministry of Industry and Information Technology, Harbin 150001, People’s Republic of China; c Collaborative Innovation Center of Extreme Optics, Shanxi University, Taiyuan, Shanxi 030006, People’s Republic of China

**Keywords:** manganese doping, local heterogeneity, piezoelectric properties, Raman spectroscopy

## Abstract

Ion doping, an effective way to modify the nature of materials, is beneficial for the improvement of material properties. Mn doping exhibits gain of piezoelectric properties in KTa_1−*x*_Nb*_x_*O_3_ (KTN). However, the impact mechanism of Mn ions on properties remains unclear. Here, the effects of Mn doping on local heterogeneity and piezoelectric properties in KTN are studied. The electric field-induced strain of Mn-doped KTN is ∼0.25% at 10 kV cm^−1^, 118% higher than that of pristine KTN. Meanwhile, as a result of Mn doping, the dielectric permittivity was tripled and the ferroelectricity was modified. The changes in A_1_(2TO), B_1_ + E(3TO) and E(4TO) vibrations characterized by Raman spectra indicate increased local polarization, weak correlation of dipoles and distorted lattices in Mn-doped KTN, respectively. First-principles calculations demonstrate stronger local heterogeneity introduced by Mn dopants, which weakens the dipole correlations and reduces domain sizes. As a result, the decreased domain sizes, combined with the larger ratio of lattice parameters *c* and *a* of the Mn-contained portion, are responsible for the higher piezoelectricity. This work reveals the impact on properties of KTN from Mn dopants and the prominent role of local heterogeneity in improving piezoelectricity, being valuable for the optimization and design of material properties.

## Abstract 2

A large enhancement (118%) of electric field-induced strain of KTa_1−*x*_Nb_*x*_O_3_ is achieved by Mn doping. The intensified local heterogeneity and modified structural features yielded by Mn dopants are demonstrated to be responsible for improved piezoelectricity. The role of Mn doping in local structures and properties of KTa_1−*x*_Nb_*x*_O_3_ crystals is revealed, providing a basis for material design and performance optimization.

## 1. Introduction   

Ion doping, which is a classic control method to change the nature of materials, has received extensive attention, especially in the field of perovskite oxides (Xu *et al.*, 2016 ▸; Li *et al.*, 2018 ▸; Oh *et al.*, 2015 ▸; Samara, 2003 ▸). Substitutions of *A*-site and *B*-site ions in perovskite *AB*O_3_ lattices, such as transition metals and rare-earth elements, tend to bring enhancements of ferroelectric, piezoelectric and dielectric properties (Zhang *et al.*, 2005 ▸; Wang *et al.*, 2017 ▸; Lin *et al.*, 2007 ▸). The positions and valence states of these dopants always influence the features of aging and anti-fatigue, which can be used to make materials more robust. Recently, Li *et al.* (2019 ▸) successfully achieved the Sm^3+^ doping of PMN-PT single crystals. The Sm^3+^ dopant modifies the local structure, largely improving the macroscopic piezoelectric performance. Ultrahigh piezoelectric coefficients (*d*_33_) of up to ∼3400 pC N^−1^ and dielectric permittivity (ɛ_33_/ɛ_0_) above 11 000 at room temperature can be achieved. Zheng *et al.* (2017 ▸) designed a new ceramic system (1−*x*)(K_1−*y*_Na*_y_*)(Nb_1−*z*_Sb*_z_*)O_3_–*x*Bi_0.5_(Na_1−*w*_K*_w_*)_0.5_HfO_3_. Multifarious dopants in the ceramic system achieve the high-temperature stability of electric field-induced strain in the range 27°C to 80°C and good fatigue properties (stable up to 10^6^ cycles) as well as an enhanced *d*_33_ of 525 pC N^−1^. Thus, ion doping plays a key role in piezoelectric and ferroelectric properties of perovskite materials.

Potassium tantalate niobate (KTa_1−*x*_Nb*_x_*O_3_, KTN), as a typical lead-free perovskite solid solution, has great electro-optic and piezoelectric properties (Chang *et al.*, 2013 ▸; Tan *et al.*, 2018*b* ▸; Lerner *et al.*, 2007 ▸). In KTN, *A*-sites are occupied by K^+^ and *B*-sites are filled by Ta^5+^ or Nb^5+^. A large number of studies have shown that doping of KTN crystals directly affects performance (Nakamura *et al.*, 2006 ▸; Lu *et al.*, 2017 ▸; Ishai *et al.*, 2004 ▸; Wang *et al.*, 2014*c* ▸). In Li^+^-doped KTN single crystals, the subwavelength anti-diffraction beam propagation and giant broadband refraction in the visible are achieved (DelRe *et al.*, 2015 ▸, 2011 ▸; Parravicini *et al.*, 2012*a* ▸,*b* ▸; Di Mei *et al.*, 2018 ▸). Using doped Ni^2+^, the band gap of KTN is narrowed, leading to enhancement of visible light absorption (Cao *et al.*, 2017 ▸). Fe^3+^ doping can promote the formation of oxygen vacancies because of valence mismatch, further controlling the defect dipoles (Cao *et al.*, 2019 ▸). Mn doping can improve the ferroelectric properties and piezoelectric properties, and is beneficial to greatly promote the anti-fatigue properties of many lead-free piezoelectric crystals and ceramics (Huang *et al.*, 2019 ▸; Wan *et al.*, 2014 ▸; Leontsev & Eitel, 2009 ▸). In addition, Mn and Fe co-doped KTN crystals can effectively reduce the recording time of electro-holography and realize high diffraction efficiency of up to 78.3% (Wang *et al.*, 2014*b* ▸). However, the physical mechanism of how such properties are attributed to doping ions is still unclear, hindering the design of material performance. Thus, deepening the study of doped KTN crystal and revealing the contribution of doping ions has become the core of properties optimization. Manganese is a typical doping element which has an obvious impact on the properties of KTN crystals. This work studies Mn-doped KTN crystals with the aim to emphasize the impact of Mn doping. Compared with pristine KTN crystals, piezoelectric, dielectric and ferroelectric properties as well as the domain structure of Mn-doped KTN crystals were characterized. The electric field-induced strain of Mn-doped KTN is largely enhanced, being nearly 0.25% under an electric field of 10 kV cm^−1^; the dielectric permittivity is tripled and the ferroelectric properties are modified in Mn-doped KTN crystals; Mn dopants reduce domain sizes. Raman spectra, which can show structural features and lattice vibration modes, are mainly used here to study the reasons why Mn doping improves the above performances. In order to reveal the contributions of ions, the angular-dependences of Raman spectra were used to indicate the symmetry of Raman tensor for characteristic vibration modes. The A_1_(2TO), B_1_+E(3TO) and A_1_(3TO) peaks exhibit the activities and intensities of local polarization. The frequency shifts of the characteristic peaks show the change of bond length. The intensity changes of these peaks reflect the alteration of polarization intensity. Furthermore, combined with first-principles calculations, the contribution of Mn doping to local structure and the effect of octahedral distortion and vacancy are speculated. The prominent impacts on domain configurations and large piezoelectricity from Mn-dopant-enhanced local heterogeneity are emphasized. This work demonstrates the role of Mn doping in the local structure and properties of KTN crystals, providing a basis for material design and performance optimization. Also, the combination of Raman spectra and first-principles calculations shows a good applicability in the field of studying the effects of ion doping.

## 2. Experimental   

### 2.1. Growth of Mn-doped KTa_1−*x*_Nb*_x_*O_3_ single crystals   

The pristine and Mn-doped KTN single crystals were grown via the top-seeded solution growth method (Tian *et al.*, 2016 ▸, 2015 ▸). The raw materials of potassium carbonate (K_2_CO_3_, 99.99%), niobium pentoxide (Nb_2_O_5_, 99.99%) and tantalum pentoxide (Ta_2_O_5_, 99.99%) powders were weighed according to the phase diagram. The mole ratio of K_2_CO_3_, Nb_2_O_5_ and Ta_2_O_5_ was 1.04:0.66:0.34, and the total weight of raw materials was 100 g. Additionally, 0.185 g manganese dioxide (MnO_2_, 99.99%) was added. Then, the mixed powders were ball-milled in ethanol for 24 h. The dried mixture was placed in a platinum crucible and calcined at 900°C for 6 h to synthesize the KTN compound. Then, the compound was melted at 1250°C. The KTN crystal was grown on a seed in the [001] direction at the specific crystallization temperature according to the phase diagram. To obtain an appropriate size of the square cross-section, the crystal growth was started at 1150°C, and then the temperature was increased rapidly. When the size of the square cross-section was miantained at 8 × 8 mm, the temperature was slowly cooled at a rate of nearly 0.6°C h^−1^ until the growth was complete. The rotation rate remained at 10 rpm and the pulling rate remained at 0.3 mm h^−1^ during the growth. Finally, the as-grown crystals were cooled to room temperature at a rate of 50°C h^−1^. The photographs of the crystal samples are shown in Fig. S1 of the supporting information.

### 2.2. Raman spectra   

The Raman spectra were measured by a Renishaw inVia confocal micro-Raman spectroscopy system using a TE air-cooled 576 × 400 CCD array with a resolution of 1 cm^−1^ at a back-scattering geometry. The geometric configurations are expressed by the Porto notation: VH represents that incident and scattered light beams have perpendicular polarizations along [100]_C_ and [001]_C_, respectively. By contrast, VV represents that the polarizations of the incident and scattered light beams are the same, both along the [100]_C_ direction. In addition, the incident light and detected scattered light are vertical with respect to the face of the polarized sample.

### 2.3. Characterizations of properties and domain configurations   

The dielectric permittivity was measured using an LCR meter (E4980A, Agilent Technologies) and applying a probing voltage (1 V). The heating rate was 0.5 K min^–1^ and the data acquisition interval was 1 K. The *P*–*E* loop and *S*–*E* loop were measured at 10 Hz using a modified ferroelectric test system (Precision Premier II, Radiant Technology Inc.). X-ray diffraction (XRD) is used to measure the lattice parameters. The XRD data were collected by a diffractometer (Empyrean, PANalytical) with a Cu *K*α radiation tube at 40 kV and 40 mA with a step size of 0.0001° at room temperature, and the contribution of Cu *K*α_2_ was removed by the MDI *Jade* software (Materials Data Inc., Livermore, CA, USA). The ferroelectric domains were observed using polarized light microscopy (Axiokop 40, Zeiss).

## 3. Results and discussion   

### 3.1. Mn-doping enhanced piezoelectric and dielectric properties   

**Figure 1:**
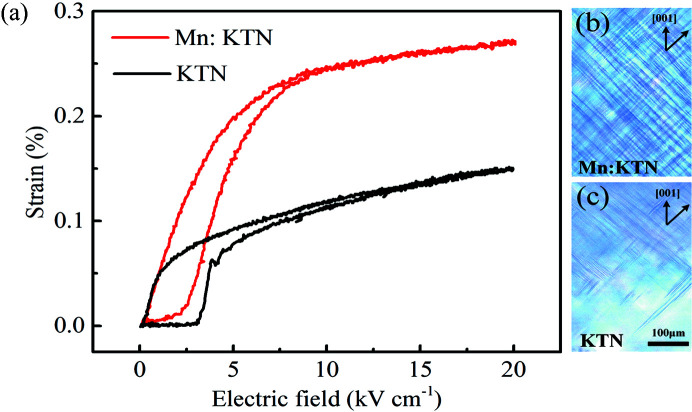
Electric field-induced strain. (*a*) Unipolar strain curves and polarized light microscopy images at 25°C of (*b*) Mn-doped KTN and (*c*) pristine KTN, respectively.

The tetragonal phase Mn-doped KTN and pristine KTN at room temperature were used in this work. Pristine KTN and Mn-doped KTN single crystals were grown via an improved top-seeded solution growth method (Tian *et al.*, 2016 ▸). The dielectric permittivity versus temperature shows that the Mn-doped KTN and pristine KTN samples have the same Curie temperature (*T*_C_ = 57°C). In order to illustrate the role of Mn dopants in piezoelectricity, the electric field-induced strains were first characterized at 25°C. Fig. 1 ▸(*a*) shows the electric field-induced strain curves (that is, *S*-*E* curves) of Mn-doped KTN and pristine KTN. It is clear that Mn doping largely enhances the electric field-induced strain. At an electric field of 10 kV cm^−1^, the strain of Mn-doped KTN can be as high as 0.25%, 118% higher than that of pristine KTN. According to the relation of the piezoelectric coefficient *d*_33_^*^ = *S*_max_/*E*_max_, where *S*_max_ and *E*_max_ are the maximum strain and electric field, respectively, *d*_33_^*^ of Mn-doped KTN is ∼2500 pC N^−1^ at 10 kV cm^−1^, which is more than double that of pristine KTN (∼1200 pC N^−1^). In particular, *d*_33_^*^ of Mn-doped KTN is up to ∼4000 pC N^−1^ under an electric field of 5 kV cm^−1^, which is more attractive and presents potential for piezoelectric applications. The domain structures, as shown in Figs. 1 ▸(*b*) and 1(*c*), manifest the reason in the mesoscopic as to why Mn doping improves *S*–*E* properties. The piezoelectricity of ferroelectrics is related to the domain configurations. Decreasing domain size always leads to higher piezoelectricity (Wada *et al.*, 2005 ▸; Hlinka *et al.*, 2009 ▸; Ahluwalia *et al.*, 2005 ▸; Yan *et al.*, 2017 ▸). The decreased difficulty of domain switching raises the electric field-induced strain.

**Figure 2:**
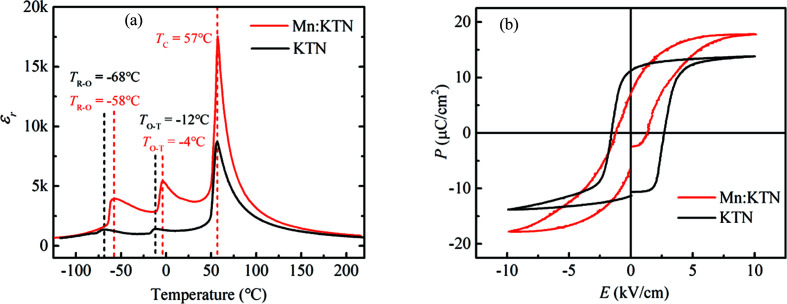
Dielectric and ferroelectric properties. (*a*) Temperature-dependences of the relative dielectric permittivity (ɛ_r_) measured at 1 kHz and (*b*) *P*-*E* loops of Mn-doped KTN and pristine KTN at 25°C.

In addition to electric field-induced strain, dielectric and ferroelectric properties are also modified by Mn doping. The temperature dependences of the relative dielectric constant ɛ_r_ of Mn-doped KTN and pristine KTN are shown in Fig. 2 ▸(*a*). The smaller domains in Mn-doped KTN tend to be easier to switch under driven fields and further yield more equivalent charge which increases the measured capacitance, contributing to the almost tripled ɛ_r_ (from ∼1300 to ∼3800) at room temperature and the stronger dielectric peak (ɛ_r_ up to ∼17 000) at *T*_C_. The *P*-*E* loops of Mn-doped KTN and pristine KTN are also very different under an electric field with a frequency of 100 Hz, as shown in Fig. 2 ▸(*b*). It is clear that the coercive field is reduced and the saturation polarization is enhanced in Mn-doped KTN. The decreasing coercive field also demonstrates the smaller driven fields for domain switching. As a result, Mn doping can alter the response behaviors of KTN single crystals to an electric field, largely enhancing the piezoelectric and dielectric properties. To understand the impact of Mn dopants on domain configurations and the micro origin of enhanced electric field-induced strain, we studied the features of local structures and their contributions to multiple properties as follows.

### 3.2. Raman spectra of Mn-doped and pristine KTN crystals   

**Figure 3:**
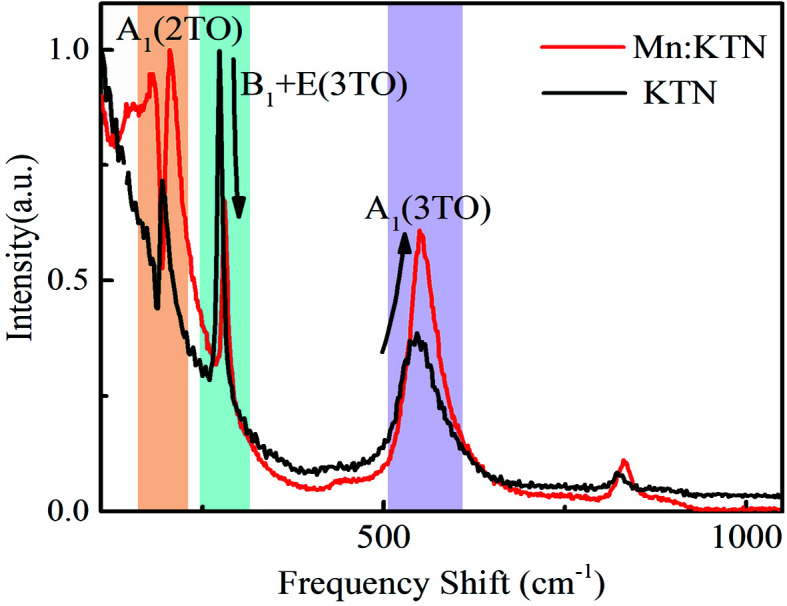
Raman spectra of Mn-doped and pristine KTN in VH configuration.

Raman spectra are an effective means of showing structural features and lattice-vibration modes. Here, Raman spectra were mainly used to indicate the effect of Mn doping. Fig. 3 ▸ exhibits the Raman spectra of Mn-doped KTN and pristine KTN in VH configuration. The marked vibration modes are related to the polar units in *AB*O_3_ perovskite (Bouziane *et al.*, 2005 ▸; Wang *et al.*, 2020 ▸, 2019 ▸, 2014 ▸ *a*; Manlief & Fan, 1972 ▸). The A_1_(2TO) mode around 200 cm^−1^ is identified as the *B*-site ions against all the oxygen ions in the NbO_6_/TaO_6_ octahedral along the direction of spontaneous polarization. The opposite motions of oxygen and *B*-site ions are generally regarded as the origin of polarization. The correlation of continuous state and discrete A_1_(2TO) mode generates Fano resonance. This Fano resonance depends on the intensity of the A_1_(2TO) mode and the degree of disorder of local dipoles. The enhancements of asymmetry and intensity of the Fano resonance after Mn doping indicate that Mn cations increase spontaneous polarization and degree of disorder of local dipoles. Hence, the enhanced spontaneous polarization after Mn doping leads to the larger saturation polarization as shown in Fig. 2 ▸(*b*). Moreover, the frequency shifts below 200 cm^−1^ are deeply influenced by translational modes of K^+^ (A-site) vibration (Sang *et al.*, 2017 ▸). The new peaks below 200 cm^−1^ indicate the breaking of translational modes of K^+^, indicating that Mn ions are likely to be doped at A-sites. This is also consistent with the change of coercive fields of *P*-*E* loops. The decreasing coercive fields are in accordance with the feature of soft doping (Berlincourt, 1992 ▸), also demonstrating that K^+^ are substituted by Mn cations with higher positive valence. The A_1_(3TO) mode in Fig. 3 ▸ is also related to the polarization of polar units (Wang *et al.*, 2019 ▸, 2020 ▸). The center of symmetry of the anion is offset because the vibration of two oxygen ions along the polar direction oppose the vibrations of the other four oxygen ions in the plane perpendicular to polar direction. The enlarged relative intensity of the A_1_(3TO) mode after doping means an increase of polarization of unit cells. The B_1_+E(3TO) mode is the sign of the wave-flapping vibration of the four oxygen ions in the plane perpendicular to spontaneous polarization (Wang *et al.*, 2019 ▸, 2020 ▸). The large intensity of the B_1_+E(3TO) mode corresponds to the strong correlation and reduced activity of local dipoles. The weaker B_1_+E(3TO) peak in Fig. 3 ▸ reveals that Mn doping promotes disorder of local dipoles.

**Figure 4:**
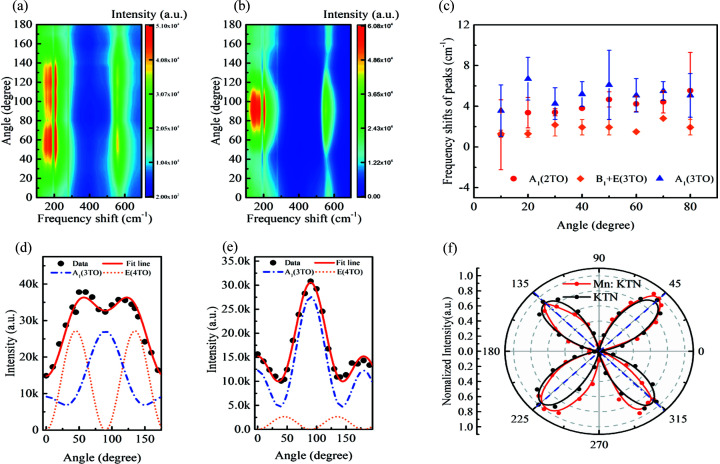
Changes of Raman spectra induced by Mn doping. Angular-dependent Raman spectra of (*a*) Mn-doped and (*b*) pristine KTN in VV configuration. (*c*) Frequency shifts of the peaks of the A_1_(2TO), B_1_+E(3TO) and A_1_(3TO) modes between KTN and Mn-doped KTN. Error bars represent the standard deviation from at least three independent measurements. Angular dependences of the A_1_(3TO) and E(4TO) modes and fitting results of (*d*) Mn-doped and (*e*) pristine KTN in the VV configuration, respectively. (*f*) Angular dependence of the A_1_(3TO) mode in the VH configuration in polar coordinates.

Different Raman active modes have the respective Raman tensors with corresponding symmetries. The angular dependences of the Raman spectra of Mn-doped and pristine KTN in VV configuration are shown in Figs. 4 ▸(*a*) and 4(*b*), respectively. The results of KTN mainly comply with the symmetry of the A_1_ mode with the maximum at 90°. Nevertheless, the Mn-doped KTN shows a distinct difference with two maximal values in the range 0° to 180°, which embodies the features of the double-degenerate E mode. The main active mode obeys the equation as follows (Fujii *et al.*, 2014 ▸)
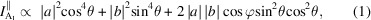


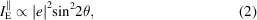
where the *a*, *b* and *e* are the elements of Raman tensor, φ is the phase retardation of the complex amplitude for *a* and *b*, and θ is the rotation angle of the sample. Using the above equations, the angular dependences of the A_1_(3TO)+E(4TO) mode of Mn-doped and pristine KTN in the range 530–565 cm^−1^ are fitted as shown in Figs. 4 ▸(*d*) and 4(*e*), respectively. Obviously, the contribution of the E mode is comparable to the A_1_ mode after Mn doping. The strengthening of E(4TO) after Mn doping is considered as that local structures tend to be orthorhombic, implying a more structural distortion. During the tetragonal–orthorhombic phase transition, E(4TO) + A_1_(3TO) evolves to B_2_(y) + A_1_(3TO). B_2_(y) has the same Raman tensor formation with E(4TO) but the space group changes from *mm*2 to 4*mm* (Zhang *et al.*, 2012 ▸). The respective contributions of E(4TO) and A_1_(3TO) are shown by Lorenz-peak fittings in Section S2 of the supporting information. The more similar the local heterogeneity is to orthorhombic symmetry, the stronger the E(4TO) peak is. Hence, Mn-doped KTN, having higher E(4TO) intensity, has larger lattice distortion. Meanwhile, the frequency shifts of the main peaks are shown in Fig. 4 ▸(*c*). All the modes have a small blue shift as a result of Mn doping, which manifest as shrinking of the lattice caused by the decreasing bond length. The angular dependence of the A_1_(3TO) mode in VH is shown in Fig. 4 ▸(*f*) to display its symmetry, which avoids the interference of the E(4TO) mode. Compared with KTN, Mn-doped KTN shows a small deviation in the angle positions of the maxima. This is because the local lattice distortions in poled samples can cause a small deviation in the local polarization relative to the poling direction, changing the angular dependence of the A_1_(3TO) mode.

### 3.3. Impact of Mn doping on local structures   

**Figure 5:**
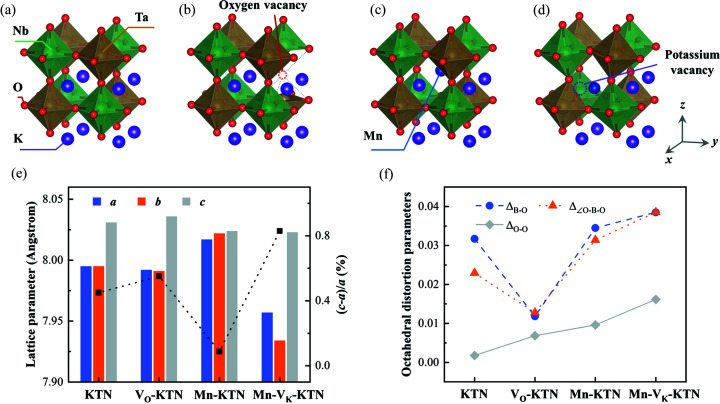
First-principles calculations for Mn-doped KTN. (*a*)–(*d*) Relaxed 2 × 2 × 2 supercell structures of pristine KTN, KTN with an oxygen vacancy (V_O_-KTN), and Mn-doped KTN without and with a potassium vacancy (Mn-KTN and Mn-V_K_-KTN), respectively. (*e*) Lattice parameters for the above four structures. (*f*) Octahedral distortions quantified by the relative changes in the average B—O bond length, O—B—O angle and O—O distance.

To further understand the contribution mechanism of Mn doping to properties, we employed first-principles calculations to explore the impact on the local structure from the doped Mn ions. Pristine KTN, KTN with an oxygen vacancy (V_O^··^_) and Mn-doped KTN without and with a potassium vacancy (V_K_′) were simulated by a 2 × 2 × 2 supercell. Please see Section S3 of the supporting information for simulation details (Kohn & Sham, 1965 ▸; Hohenberg & Kohn, 1964 ▸; Giannozzi *et al.*, 2009 ▸; Takenaka *et al.*, 2017 ▸; Tan *et al.*, 2018*a* ▸; Perdew *et al.*, 1996 ▸; Grinberg *et al.*, 2000 ▸). The relaxed structures are shown in Figs. 5 ▸(*a*)–5(*d*), of which the structural parameters are shown in Fig. 5 ▸(*e*) and Table S1 of the supporting information. The undoped KTN exhibits a tetragonal crystal structure, and the lattice parameters of the 2 × 2 × 2 supercell are calculated to be *a* = 7.995 and *c* = 8.031 Å which differ only by less than 5% from the XRD results (see Section S1 of the supporting information). For the other three structures in Fig. 5 ▸, because of the limited number of atoms, our simulations have a much higher dopant/vacancy concentration (12.5 mol% in the supercell) than the as-grown crystals. Thus, the relative variations in structure between the pristine and doped crystals are concerned here. Moreover, considering the trends of coercive fields and Raman characteristic peaks after Mn doping, we substituted an Mn ion in the A site. As shown in Fig. 5 ▸(*e*), compared with pristine KTN, the lattice constants change only a little for KTN with V_O^··^_, but Mn-contained structures show great lattice constant variations which agree with the XRD results (see Section S1 of the supporting information). However, when only adding an Mn cation, the relative trends in the value of (*c* − *a*)/*a* and the volume of the unit cell differ from experimental results. Additionally, V_O^··^_ do not yield notable changes of volume, as shown in Fig. 5 ▸(*e*). Therefore, the V_K′_ is added as shown in Fig. 5 ▸(*d*). We speculate that Mn-doped KTN with V_K′_ is probably closer to reality, because an Mn cation usually has a valence greater than +1 so that V_K′_ is beneficial to balance the charge in the Mn-doped structure. Also, the above experimental results show that Mn-doped KTN with V_K′_ is more qualitatively similar to our as-grown crystal. That is, Mn doping enhances the electric field-induced strain indicating a larger (*c* − *a*)/*a*; the XRD results (Fig. S1) embody a decrease in the volume of the Mn-doped KTN unit cell.

Furthermore, to reveal the local distortions in this class of materials, we studied the octahedral distortions using the structural parameters from these relaxed structures. The distortions in each octahedron are estimated by bond-length distortion Δ_B–O_, bond-angle distortion Δ_∠O–B–O_ and edge-length distortion Δ_O–O_, as shown in Fig. 5 ▸(*f*). The definitions of these distortion parameters are shown in Section S4 of the supporting information (Ertl *et al.*, 2002 ▸; Reyes-Martinez *et al.*, 2020 ▸). Mn doping, especially in the Mn-doped KTN with V_K′_, significantly increases octahedral distortion, highlighting its impact on local structures. Not only does the large (*c* − *a*)/*a* value of the Mn-doped structure with V_K′_ reveal the origin of high electric field-induced strain in Mn-doped KTN single crystals, but also the octahedral distortions manifest the micro-mechanisms of Raman spectra evolution caused by Mn doping. The increased difference between lattice parameters *a* and *b* of Mn-V_K_-KTN, as shown in Fig. 5 ▸(*e*), make local heterogeneity close to the orthorhombic phase. The enhanced octahedral distortions can strengthen E(4TO) intensity, which coincides with the Raman spectrum [orange dotted line in Fig. 4 ▸(*d*)]. Moreover, the distortions increase local heterogeneity, broadening the distribution of orientations of local polarizations. This kind of structural change can induce deviation of angular dependences of Raman signal, such as A_1_(3TO) in Fig. 4 ▸(*f*). In addition, the potassium vacancy can buffer the geometric deformation due to the extended free space, which may also be one of the reasons why the coercive field is reduced in Mn-doped KTN [Fig. 2 ▸(*b*)].

The DFT results are consistent with the structural characteristics illustrated by the Raman spectra, showing the impact on local structures from Mn doping. Both the simulated and the spectroscopically measured results indicate the enhanced local distortions in Mn-doped KTN. The increase in lattice distortions can directly yield stronger local heterogeneity. The correlation of adjacent local dipoles is weakened by the enlarged local heterogeneity, so that the domain sizes are reduced and the density of the domain walls is raised as shown in Figs. 1 ▸(*b*) and 1(*c*). Decreased domain size, combined with the larger (*c* − *a*)/*a* of the Mn-contained portion, are responsible for the higher piezoelectricity of Mn-doped KTN (that is, the electric field-induced strain and *d*_33_^*^). The physics mechanism of decreased domains in Mn-doped KTN is different from that of Mn-doped KNN. The local heterogeneity is considered responsible for smaller domains in Mn-doped KTN. However, the pinning effect caused by 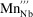 − 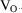 defect dipoles increases the density of domain walls (Hu *et al.*, 2020 ▸). Moreover, the large local structural heterogeneity, conducing to a flattened thermodynamic energy profile, is advantageous to promote the dielectric permittivity, which is consistent with Fig. 2 ▸(*a*). Hence, the local heterogeneity strengthened by Mn dopants makes a prominent role in the optimization of properties for KTN, which is the same as the micro origin of giant piezoelectricity of Sm-doped Pb(Mg_1/3_Nb_2/3_)O_3_-PbTiO_3_ single crystals. Based on this principle, we speculate that improving local heterogeneity by ion doping should be an efficient way to optimize piezoelectricity of perovskite crystals. Importantly, constructing local heterogeneity through appropriate ion doping, such as Mn doping in KTN or K_1−*y*_Na*_y_*NbO_3_ systems, is much more likely to facilitate lead-free piezoelectric materials with more application potential.

## 4. Conclusions   

We have studied the impact of Mn doping in KTN single crystals on performance and local structures. The piezoelectric, dielectric and ferroelectric properties were characterized, showing the gain effect of Mn doping. Under the action of Mn doping, the electric field-induced strain was largely strengthened up to ∼0.25% at an electric field of 10 kV cm^−1^, being 118% higher than that of pristine KTN; the ɛ_r_ at *T*_C_ almost doubled (up to 17 000); the saturation polarization was enhanced, while the coercive field was reduced. The Raman spectra and first-principles calculations were used to study the local structure features and origins of the enhanced performances. The intensity of the A_1_(2TO) vibration mode related to the polarization of unit cells is increased, indicating raised local polarization caused by Mn doping. The low intensity of the B_1_ + E(3TO) vibration mode manifests the weak correlation of dipoles, highlighting the reduced coercive field caused by Mn doping. The more drastic the vibration of the E(4TO) mode means that the local structure is more similar to the orthorhombic phase, revealing that Mn doping intensifies local distortions (that is, local heterogeneity), also demonstrated by DFT calculations. The increase of lattice distortions conveys the stronger local heterogeneity, being able to diminish dipole correlations and then decrease the domain sizes. The characteristics of smaller domains that can easily be driven by electric field are beneficial to improve piezoelectricity. Moreover, in terms of changing trends of lattice volumes, we speculate that V_K′_ occurs with Mn doping, enlarging the local distortions and co-contributing to the locally large (*c* − *a*)/*a* value. As a result, the decreased domain sizes, combined with the larger (*c* − *a*)/*a* values of the Mn-contained portion, indicate the origin of enhanced electric field-induced strain. Our work reveals the gain mechanisms on properties of KTN single crystals from Mn dopants, further emphasizing the prominent role of local heterogeneity in improving piezoelectricity. This work provides an effective foundation to study the features of structures and performances in ion-doped perovskite materials, being beneficial for the optimization and design of functional material properties.

## Supplementary Material

Details of experiments and simulations. DOI: 10.1107/S2052252521000890/yc5028sup1.pdf
