# Supplementary material for: Manganese-doping enhanced local heterogeneity and piezoelectric properties in potassium tantalate niobate single crystals
Source: IUCrJ. 2021 Feb 24;8(Pt 2):319–26. doi: 10.1107/S2052252521000890 (PMC7924224; doi:10.1107/S2052252521000890)
Supplement: Supplementary file 1 [file m-08-00319-sup1.pdf]

IUCrJ

Volume 8 (2021)

Supporting information for article:

Manganese doping enhanced local heterogeneity and piezoelectric properties in potassium tantalate niobate single crystals

Yu Wang, Peng Tan, Xiangda Meng, Zhongxiang Zhou, Xiaolin Huang, Chengpeng Hu, Fei Huang, Jing Wang and Hao Tian

S1. X-ray diffraction measurements

The X-ray (λ = 1.5406 Å) diffraction patterns were collected by a diffractometer (Empyrean, PANalytical) with Cu-Kα radiation, as shown in Fig. S1. The lattice parameters are estimated as $a = b = 3.992$ Å and $c = 4.007$ Å for the pristine potassium tantalate-niobate single crystal ($KTa_{1-x}Nb_xO_3$, abbreviated as KTN). The lattice of Mn doped KTN are smaller than the pristine KTN, which indicates the shrinking of unit cells.

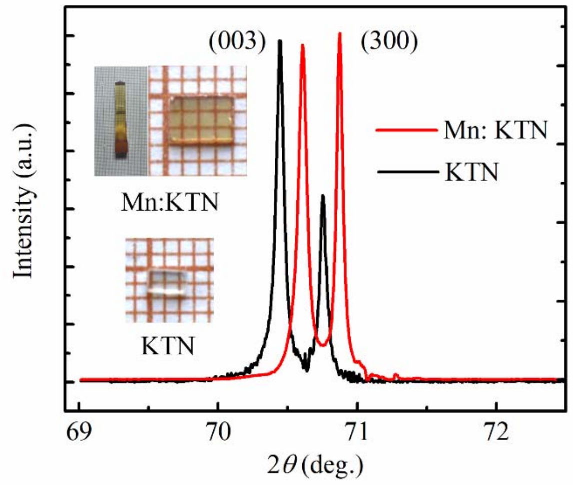

Figure S1 Room-temperature X-ray diffraction patterns for Mn doped KTN and pristine KTN single crystals. The insets show the photographs of samples and whole Mn doped KTN crystal used here.

S2. Lorenz-peak fittings of Raman spectra

The normalized Raman spectra peak fittings of pristine KTN and Mn doped KTN are shown in Fig. S2. The peak at 530cm^{-1} is identified to the E(4TO) mode of tetragonal phase, which evolves to the B_2(y) mode in orthorhombic phase. It relates to the difference between lattice parameters a and b. The E(4TO) mode in KTN is small due to $a = b$ in tetragonal phase. The Mn dopants largely enhance the intensity of E(4TO) mode, indicating the increased difference between a and b (that is, the enlarged local distortion). The peak of pristine KTN at 608cm^{-1} is a second order mode.

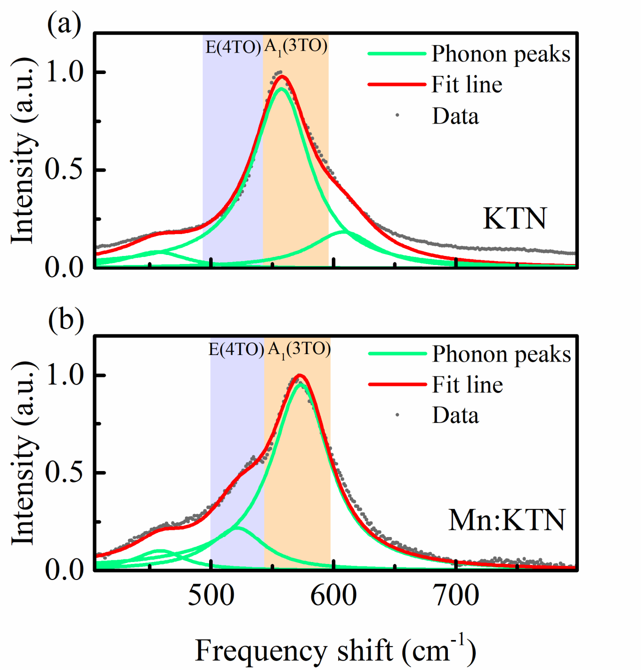

Figure S2 Lorenz-peak fittings in range of 400 cm^{-1} to 700 cm^{-1} of normalized Raman spectra in geometric VV configuration for (a) pristine KTN and (b) Mn-doped KTN, respectively. The characteristic peaks of E(4TO) and A_1(3TO) are exhibited in violet and orange regions, respectively.

S3. Computational details

Pristine KTN, KTN with an oxygen vacancy ($V_O^{\bullet\bullet}$), and Mn-doped KTN without and with a potassium vacancy (V_K') were simulated by a 2 × 2 × 2 supercell, containing 40, 39, 40, and 39 atoms, respectively (Figure 4 of the main text). All calculations are performed based on Density Functional Theory (DFT) with a plane-wave basis set using the Quantum Espresso software package.(Kohn *et al.*, 1965; Hohenberg *et al.*, 1964; Giannozzi *et al.*, 2009) This model is large enough to capture the local structure characteristics.(Li *et al.*, 2019; Takenaka *et al.*, 2017; Tan & Takenaka *et al.*, 2018) Also, we used a $KTa_{0.50}Nb_{0.50}O_3$ composition that allowed for a supercell size feasible for DFT calculations and was only a few percent larger in Nb content than the as-grown crystal ($KTa_{0.56}Nb_{0.44}O_3$). The generalized gradient approximation (GGA) with the Perdew-Burke-Ernzerhof (PBE) exchange correlation (XC) functional have been used.(Perdew *et al.*, 1996) All atoms are represented by

norm-conserving pseudopotentials generated with the Opium package,(Opium-Pseudopotential Generation Project, Grinberg *et al.*, 2000) which treated the K 3s3p3d4s4p, Mn 3s3p3d4s, Ta 5s5p5d6s, Nb 4s4p4d5s, and O 2s2p states as valence electrons. A kinetic energy cutoff of 680 eV for wavefunctions is adopted and the charge cutoff is four times larger. For all structures, a 4 × 4 × 4 Monkhorst-Pack k-mesh is used and the structures are optimized until the ionic forces on each atom are below 20 meV/Å. In all calculations, structures are fully relaxed permitting their atomic coordinates and lattice vectors to relax, and all the impurity and defects were set to be neutral. The structural parameters of relaxed 2 × 2 × 2 supercells are listed in Table S1.

Table S1 Supercell parameters

Structure	*a* (Å)	*b* (Å)	*c* (Å)	*α* (°)	*β* (°)	*γ* (°)
KTN	7.995	7.995	8.031	90.0	90.0	90.0
KTN with a $V_O^{\bullet\bullet}$	7.992	7.991	8.036	89.9	90.1	90.0
Mn-doped KTN	8.017	8.022	8.024	90.3	89.9	89.9
Mn-doped KTN with a V_K'	7.957	7.934	8.023	90.2	90.4	89.9

S4. Calculation of octahedral distortions

Here, we study the octahedral distortions (changes in the bond lengths and angles) using the structural parameters from the fully relaxed structures. The distortions in BO_6 octahedron are estimated by bond-length distortion $\Delta_{B\text{–}O}$, bond-angle distortion $\Delta_{\angle O\text{–}B\text{–}O}$, and edge-length distortion $\Delta_{O\text{–}O}$.(Ertl *et al.*, 2002; Reyes-Martinez *et al.*, 2020) The definitions for these distortion parameters are listed as follows:

$$\Delta_{B-O} = \left\{ \sum_{i=1}^{n_B} \left[\left(\sum_{j=1}^{n_{B-O}} |l_j - l_0| \right) / (n_{B\text{-}O} \cdot l_0) \right] \right\} / n_B$$

$$\Delta_{\angle O-B-O} = \left\{ \sum_{i=1}^{n_B} \left[\left(\sum_{j=1}^{n_{\angle O-B-O}} |\theta_j - \theta_0| \right) / (n_{\angle O-B-O} \cdot \theta_0) \right] \right\} / n_B$$

$$\Delta_{O-O} = \left\{ \sum_{i=1}^{n_B} \left[\left(\sum_{j=1}^{n_{O-O}} |d_j - d_0| \right) / (n_{O-O} \cdot d_0) \right] \right\} / n_B$$

where l_i, θ_j, and d_j are the B–O bond length, ∠O–B–O bond angle, and O–O distance, respectively; l_0, θ_0, and d_0 are the average B-O bond length, average ∠O–B–O bond angle, and average O–O distance in the i-th octahedra; and n_B is the number of B-site cations in the structure. For $\Delta_{\angle O\text{–}B\text{–}O}$ and $\Delta_{O\text{–}O}$, only O–O distances and ∠O–B–O angles between neighboring atoms in each octahedra are taken into account.

References

Ertl, A., Hughes, J. M., Pertlik, F., Foit, F. F., Wright, S. E., Brandstatter, F. & Marler, B. (2002). *Can. Mineral.*, **40**, 153.

Giannozzi, P., Baroni, S., Bonini, N., Calandra, M., Car, R., Cavazzoni, C., Ceresoli, D., Chiarotti, G. L., Cococcioni, M., Dabo, I., Corso, A. D., De Gironcoli, S., Fabris, S., Fratesi, G., Gebauer, R., Gerstmann, U., Gougoussis, C., Kokalj, A., Lazzeri, M., Martin-Samos, L., Marzari, N., Mauri, F., Mazzarello, R., Paolini, S., Pasquarello, A., Paulatto, L., Sbraccia, C., Scandolo, S., Sclauzero, G., Seitso-nen, A. P., Smogunov, A., Umari, P. & Wentzcovitch, R. M. (2009). *J. Phys.-Condens. Mat.*, **21**, 395502.

Grinberg, I., Ramer, N. J. & Rappe, A. M. (2000). *Phys. Rev. B*, **62**, 2311.

Hohenberg, P. & Kohn, W. (1964). *Phys. Rev.*, **136**, B864.

Kohn, W. & Sham, L. J. (1965). *Phys. Rev.*, **140**, A1133.

Li, F. Cabral, M. J., Xu, B., Cheng, Z., Dickey, E. C., LeBeau, J. M., Wang, J. L., Luo, J., Taylor, S., Hackenberger, W., Bellaiche, L., Xu, Z., Chen, L. Q., Shrout, T. R. & Zhang, S. J. (2019). *Science*, **364**, 264.

Opium - Pseudopotential Generation Project, http://opium.sourceforge.net

Perdew, J. P., Burke, K. & Ernzerhof, M. (1996). *Phys. Rev. Lett.*, **77**, 3865.

Reyes-Martinez, M. A., Tan, P., Kakekhani, A., Banerjee, S., Zhumekenov, A. A., Peng, W., Bakr, O. M., Rappe, A. M. & Loo. Y.-L. (2020). *ACS Appl. Mater. Inter.*, **12**, 17881.

Takenaka, H., Grinberg, I., Liu, S. & Rappe, A. M. (2017). *Nature*, **546**, 391.

Tan, H., Takenaka, H., Xu, C., Duan, W., Grinberg, I. & Rappe, A. M. (2018). *Phys. Rev. B*, **97**, 174101.
